# Natural Umbilicoplasty Without Visible Scarring: An Easily Taught and Reproducible Technique

**DOI:** 10.1093/asjof/ojad027.017

**Published:** 2023-04-14

**Authors:** Joshua Henderson, Luis Quiroga, Jack Gelman

**Affiliations:** West Virginia University, Morgantown, WV; West Virginia University, Morgantown, WV; West Virginia University, Morgantown, WV

## Abstract

**Goals/Purpose:**

The umbilicus is a focal point in an abdominoplasty, but the variety of published techniques can create confusion when deciding which incision is optimal for inset. Recently, an excellent technique was described that results in a scarless caudal umbilicus with superior hooding (2022 Samargandi). A similar technique has been used by this abstract’s senior author (J.G.) for over 20 years and has subsequently been implemented in our residency training. The inset is accomplished through an inverted-V incision in the abdominoplasty flap. The technique avoids excessive vertical tension on the abdominoplasty closure, a complicating factor of aggressive abdominoplasty advancement that can lead to vascular compromise in the inferomedial aspect of the flap. Insetting the umbilicus prior to resection of this inferior excess can allow for greater advancement of the abdominoplasty and decreased risk of flap over-resection.

**Methods/Technique:**

The umbilical inset through an inverted-V incision in the abdominoplasty flap has been performed in all the senior author’s (J.G.) patients over the past 20 years. Following inferior wedge resection of the native umbilical stalk, the apex of the inferiorly based (inverted-V) flap is sutured to the dermis of the umbilical stalk and to the rectus fascia. The superolateral margins of the native umbilicus are approximated to the abdominoplasty skin. The sole deep suture inferomedially tethers the most visible aspect of the umbilicoplasty to the abdominal wall. The inverted-V incision in the abdominoplasty flap prior to low transverse closure increases the vertical tension on the flap and enables a safe resection of the inferior excess tissue. This technique has been taught to all residents in our training program and quickly incorporated in our abdominoplasty closures.

**Results/Complications:**

Incising the inverted-V in the abdominoplasty flap eases tension on the low transverse wound closure. All residents have been able to independently perform this technique. Among the patients who have undergone this technique, none have experienced necrosis of the inferior aspect of the abdominoplasty flap or of the umbilical stalk base. As gravity encourages relaxation of the abdominoplasty skin, the superior aspects of the umbilicoplasty are overshadowed by the resulting superior hooding. This technique yields a natural-appearing umbilicus with adequate depth and a camouflaged inferior inset. Our patients frequently comment on the “untouched” appearance of their umbilici. No patients have required revisions of the umbilical inset, and none have experienced necrosis of the inferomedial aspect of the abdominoplasty flap. Residents have reported quick integration and predictable umbilical positioning with this technique.

**Conclusion:**

The technique is easy to teach and can be rapidly incorporated by surgeons in practice, as well as in training. We are excited to see this technique having recently been described and feel strongly that its widespread adoption should be entertained. The inset during abdominoplasty flap advancement, prior to final resection of the inferior excess skin, can simplify decision-making for where to position the umbilicus and how much inferior excess tissue to resect. In addition to realizing the aesthetic superiority of this umbilicoplasty technique, it is important to proclaim the benefit of insetting prior to final abdominoplasty closure and the intuitive sense with which the new position of the umbilicus can be determined.

